# A three-dimensional model of the human blood-brain barrier to analyse the transport of nanoparticles and astrocyte/endothelial interactions

**DOI:** 10.12688/f1000research.7142.2

**Published:** 2016-01-21

**Authors:** Peddagangannagari Sreekanthreddy, Radka Gromnicova, Heather Davies, James Phillips, Ignacio A. Romero, David Male

**Affiliations:** 1 Department of Life, Health and Chemical Sciences, The Open University, Milton Keynes, MK7 6AA, UK

**Keywords:** in vitro model, blood-brain barrier, three-dimensional, endothelium, astrocytes, nanoparticles

## Abstract

The aim of this study was to develop a three-dimensional (3D) model of the human blood-brain barrier *in vitro*, which mimics the cellular architecture of the CNS and could be used to analyse the delivery of nanoparticles to cells of the CNS. The model includes human astrocytes set in a collagen gel, which is overlaid by a monolayer of human brain endothelium (hCMEC/D3 cell line). The model was characterised by transmission electron microscopy (TEM), immunofluorescence microscopy and flow cytometry. A collagenase digestion method could recover the two cell types separately at 92-96% purity.  Astrocytes grown in the gel matrix do not divide and they have reduced expression of aquaporin-4 and the endothelin receptor, type B compared to two-dimensional cultures, but maintain their expression of glial fibrillary acidic protein. The effects of conditioned media from these astrocytes on the barrier phenotype of the endothelium was compared with media from astrocytes grown conventionally on a two-dimensional (2D) substratum. Both induce the expression of tight junction proteins zonula occludens-1 and claudin-5 in hCMEC/D3 cells, but there was no difference between the induced expression levels by the two media. The model has been used to assess the transport of glucose-coated 4nm gold nanoparticles and for leukocyte migration. TEM was used to trace and quantitate the movement of the nanoparticles across the endothelium and into the astrocytes. This blood-brain barrier model is very suitable for assessing delivery of nanoparticles and larger biomolecules to cells of the CNS, following transport across the endothelium.

## Introduction

The human blood brain barrier presents a major challenge for the pharmaceutical industry, and a number of cell culture systems have been developed which model different aspects of the barrier ( Naik & Cucullo, 2012; Ogunshola, 2011). *In vivo*, the barrier is formed by specialised brain endothelial cells that have continuous tight junctions, low pinocytotic activity and a set of enzymes and ABC-transporters that metabolise or exclude many xenobiotics ( Sarkadi *et al.*, 2006; Wolburg & Lippoldt, 2002). The function and structure of the brain endothelium is supported by astrocytes; astrocytic foot processes ensheath the abluminal side of the endothelium and form a double basal lamina, with the endothelium ( Abbott *et al.*, 2010). Several tissue culture models of the barrier have been developed and each one has some of the characteristics seen *in vivo*, although the barrier is generally not as tight as *in vivo* ( Butt *et al.*, 1990). Hence, each model is suitable for specific purposes, although none of the models is ideal for all applications ( Naik & Cucullo, 2012). One aim of this study was to develop a model of the human blood-brain barrier, which could be used to study nanoparticle movement across brain endothelium and localisation in glial cells. This model might also be used to study interactions between brain endothelium and astrocytes.

One problem in the development of barrier models is the limited availability of primary human brain endothelium, although this has been partly compensated by the development of stem-cell derived brain endothelium ( Lippmann *et al.*, 2012). Primary endothelium at low passage has high resistance tight junctions, and expresses appropriate transporters, but the barrier phenotype tends to decline in extended culture ( Lyck *et al.*, 2009). The availability of the human brain endothelial line hCMEC/D3 ( Weksler *et al.*, 2005) has been used to develop several *in vitro* models ( Weksler *et al.*, 2013). These cells do not form a strong barrier for molecules <4kDa under static conditions, although the transendothelial electrical resistance (TEER) is enhanced in flow-based conditions ( Cucullo *et al.*, 2008).

Barrier models which include human astrocytes have generally cultured the endothelium on the upper surface of filters in transwell inserts, and the astrocytes are either cultured on the lower surface of the filter or on the base of the culture well ( Naik & Cucullo, 2012). As the pore area is generally <20% of the filter area and with a filter being 10–30μm thick, there is little opportunity for contact between the astrocytes and the endothelium as occurs *in vivo*. Moreover, growing astrocytes on a two-dimensional surface (2D-astrocytes) can affect their phenotype ( Maltman & Przyborski, 2010). Recently collagen gels containing different cell types have been used to model the three-dimensional (3D) environment found in tissues *in vivo* ( Lee *et al.*, 2008). Rat astrocytes cultured in 3D matrices are less reactive than 2D-astrocytes ( East *et al.*, 2009). They have a different morphology, slower growth rate and fewer stress fibres. 2D-astrocytes spontaneously become reactive ( Holley *et al.*, 2005; Wu & Schwartz, 1998) and may secrete inflammatory cytokines and angiogenic factors, which interfere with the maintenance of the endothelial barrier. In addition, astrocytes grown on rigid 2D surfaces are subject to different positional cues to cells in a 3D matrix. For these reasons, 2D-astrocytes do not reflect the phenotype of astrocytes *in vivo*. In the model developed here, human astrocytes were cultured in a 3D collagen hydrogel, overlaid with a human brain endothelial cell line forming a monolayer on the gel surface. The arrangement allows contact between the endothelium and astrocytes and free movement of larger biomolecules and nanoparticles between the basal surface of the endothelium and the astrocytes in the underlying gel.

## Materials and Methods

### Endothelial and astrocyte cultures

Human foetal cortical astrocytes at passage 1, were obtained from ScienCell Research Laboratories (Carlsbad, CA, USA) and were maintained on collagen type-I coated tissue culture dishes in human astrocyte medium (ScienCell Research Laboratories, Carlsbad, CA, USA) including 2% foetal bovine serum and the recommended growth supplements as per the manufacturer. Analysis of these cultures by immunofluorescence indicated that 29.2–46.9% of cells were GFAP ^+^ at the start of the cultures and approximately 90% of the cells were S100β ^+^, indicating that the majority of cells were astrocytes. Fibroblasts (TE7 ^+^) constituted 1.7–2.8% of the cells at passages 2–4. Oligodendrocytes (O1 ^+^) and microglia (CD68 ^+^) were not detectable in the astrocyte cultures.

The human cerebral microvascular endothelial cell line hCMEC/D3 ( Weksler *et al.*, 2005) at passage 24–30 was cultured on collagen-coated flasks or tissue culture inserts in EBM-2 medium (Lonza, Basel, Switzerland) supplemented with 2.5% foetal bovine serum, hydrocortisone, VEGF, epidermal growth factor (EGF), insulin-like growth factor I (IGF-I), human fibroblast growth factor (FGF), ascorbic acid and gentamicin sulphate according to the manufacturer’s formulation. All cells were cultured at 37°C in 100% humid air containing 5% CO _2_, unless otherwise indicated. The medium was changed every 2–3 days.

### 3D-astrocyte cultures and 3D-astrocyte-endothelial co-cultures

Collagen gels containing 0.5 × 10 ^6^ astrocytes per ml were prepared in 24-well plates, with an initial volume of 450μl cellular collagen gel per well. Gels were composed of a 10% cell suspension of human astrocytes, passage 2–4, in DMEM (Gibco, Thermo Fisher Scientific, UK), 10% 10× minimum essential medium (MEM; Sigma, UK) and 80% type I rat tail collagen (2.5 mg/ml; First Link, Wolverhampton, UK). The collagen was diluted from a 5mg/ml 0.6% acetic acid stock using water, then mixed with MEM and neutralised using sodium hydroxide (assessed by colour change of the phenol red indicator to orange-red), then the mixture was added to the cell suspension and mixed to ensure even distribution of cells before transferring to the 24-well plates or transwell inserts. Gelation took approximately 10 min at 37°C and then astrocyte medium was added. In preliminary experiments the astrocyte density was varied between 0.25 × 10 ^6^ astrocytes/ml and 2.0 × 10 ^6^ astrocytes/ml. At higher densities the astrocytes caused the gel matrix to contract and detach from the sides of the wells. Therefore, the optimal density for astrocytes was established as 0.5 × 10 ^6^ astrocytes/ml for cultures of ≤7 days. If the gels were compressed (see below), or for shorter duration cultures, cell densities up to 1.5 × 10 ^6^ astrocytes/ml could be used.

For astrocyte/endothelial co-cultures, astrocyte-containing gels were established as described above, either in 24-well plates or 1cm inserts, containing 0.5 × 10 ^6^ astrocytes per ml (uncompressed gel) or 1.2 × 10 ^6^ astrocytes per ml (compressed gel). After gelation astrocytes were cultured for 2h in astrocyte medium. Some of the gels were then stabilised (compressed) using RAFT™ absorbers (TAP Biosystems, Royston, UK – now available from Lonza, UK) for 15 min to remove fluid and reduce gels to less than 10% of their original volume. Uncompressed gels were 1–2mm thick: compressed gels were <0.1mm thick. The gels were maintained for a further 24h in astrocyte medium before being overlaid with hCMEC/D3 cells at a density of 60,000 cells/cm ^2^. The co-cultures were incubated at 37°C for 3 days in EGM2-MV medium, before characterisation or use in transcytosis experiments.

### Immunofluorescence *in situ*

The details of antibodies used for immunofluorescence and flow cytometry are given in Table 1. 3D cultures were stained by similar methods to conventional 2D cultures, except that the wash times were extended. The gels were rinsed with Hank’s balanced salt solution (HBSS) without Ca ^++^/Mg ^++^, fixed in 4% fresh paraformaldehyde in phosphate buffered saline (PBS) for 45 min and washed for 30 min in three changes of PBS. The gels were then detached from the wells, incubated with 0.05% w/v saponin in PBS and 5% normal goat serum for 1h and washed 3× in PBS before incubation overnight in primary antibody. (A matched isotype control was used as negative control for the mouse monoclonal antibodies: no primary antibody was used as negative for rabbit antibodies.) Gels were washed for 1h with 4 changes of PBS before incubation with secondary antibodies and 1μg/ml Hoechst 33259 for 90 min. Secondary antibodies were 10μg/ml Alexafluor-488 or -555 conjugated goat antibodies to mouse or rabbit IgG (Invitrogen Ltd). Finally the gels were washed for 6h with 12 changes of PBS and imaged using a Leica TCS SP5 confocal microscope (Leica, Germany). From the z-stack of images, a maximal intensity projection image was generated. In the case of 2D cultures, for comparison of treatments, ten images were captured in randomly selected fields using an Olympus BX61 microscope. Fluorescence intensity was analysed using ImagePro software (MediaCybernetics UK, v8) and the percentage of pixels exceeding a threshold pixel-density was measured. The threshold value was set according to the background intensity and this value was constant across all images in each experiment. For analysis of zonula occludens-1 (ZO-1), nuclear and perinuclear staining was excluded from the analysis whereas for claudin-5 (CLDN5), the intensity across the whole cell was measured. In each experiment, the results were normalised to the reference treatment, by dividing the stained area of treated cells by the stained area of control cells. The normalised values from different experiments were then combined to give mean fluorescence intensity ±SEM.

**Table 1.** Antibodies.

Antibody*	Usage	Conc.	Supplier
Aquaporin-4 Rabbit	Primary, indirect IF	2μg/ml	Santa Cruz Biotechnology Inc. #sc-20812
Claudin-5 Mouse IgG	Primary, indirect IF	2.5μg/ml	Invitrogen Ltd #35-2500
EDNRB Rabbit	Primary, indirect IF	8μg/ml	Alomone labs #AER-002
ZO-1 Rabbit	Primary, indirect IF	2.5μg/ml	Zymed #61-7300
P-gp Mouse IgG	Primary, indirect IF	5μg/ml	Kamiya Biomedical Co. #MC012
GFAP Mouse IgG	Primary, indirect IF	2.4μg/ml	Sigma Aldrich Inc. # G3893
S100β Rabbit	Primary, indirect IF	1/200	Abcam #ab52642
TE7 Mouse IgG	Primary, indirect IF	0.67μg/ml	Millipore (UK) Ltd #CBL271
O1 Mouse IgM	Primary, indirect IF	1μg/ml	R&D systems #MAB1327
CD68 Mouse IgG	Primary, indirect IF	2μg/ml	Dako (UK) Ltd #F7135
PECAM1 Mouse IgG	Primary, indirect IF	10μg/ml	R&D systems #BBA7
PECAM1-PerCP- eFluor710	Direct IF	1.25μg/ml	eBioscience Ltd #46-0319-42
Anti-mouse-IgG- AlexaFluor 488	Secondary, indirect IF	10μg/ml	Invitrogen Ltd #A11001
Anti-mouse-IgG- AlexaFluor 555	Secondary, Indirect IF	10μg/ml	Invitrogen Ltd #A21422
Anti-mouse-IgM	Secondary, Indirect IF	5μg/ml	AbD Serotec #MCA199F
Anti-rabbit-IgG AlexaFluor 488	Secondary, Indirect IF	10μg/ml	Invitrogen Ltd #A11008

* All mouse primary antibodies are monoclonal. All others are polyclonal.

### Cell recovery and flow cytometry

To recover cells, the gels were subjected to collagenase digestion. 3D gel cultures of astrocytes were rinsed with HBSS and incubated for 45 min at 37°C with 0.25% collagenase, 10μg/ml DNAse I and 0.147μg/ml TLCK hydrochloride (Tosyl-Lys-chloromethylketone.HCl) in DMEM. Digestion was terminated by addition of 20mM EDTA, and cells recovered by centrifugation at 320g for 5 min. To isolate hCMEC/D3 cells from co-cultures a two-step collagenase digestion was developed. The gels were first treated as above for 15 min and the hCMEC/D3 monolayer detached from the gel surface intact and placed in 0.25% trypsin-EDTA to dissociate the cells (step-1). The collagenase digestion of the gel was continued for a further 30 min in order to recover the astrocytes (step-2).

The two-step collagenase digest for recovering cell populations was validated by measuring the purity of the astrocyte fraction after co-culture. Endothelial cells (hCMEC/D3) were pre-labelled with a fluorescent tracker, 5-chloromethylfluorescein diacetate (CMFDA), and cultured with the astrocytes for 7 days. The endothelial monolayer was removed and the astrocytes recovered and analysed by flow cytometry (see below). The level of contamination of the astrocytes by endothelium, measured by PECAM-1 staining (endothelial marker) was in the range 5–8%, in different experiments (n=3). Median fluorescence of astrocytes in monoculture in these FACS conditions was 4.7 units, and that of astrocytes isolated from co-cultures was 5.0 units. Isolated endothelium had a median fluorescence of 187 units.

Isolated populations of endothelium or astrocytes were characterised by flow cytometry. Endothelium was labelled with monoclonal antibody to PECAM-1, directly conjugated to PerCP-eFluor-710 for 30 min at 4°C in diluent containing 0.1% NaN _3_ and 0.1% BSA in PBS. For indirect immunofluorescence, cells were trypsinised, washed and fixed in 4% paraformaldehyde. After two washes, cells were incubated with primary antibodies for 1h at room temperature in PBS containing 0.1% BSA and 2.5% normal goat serum. Control staining was carried out simultaneously with a corresponding matched isotype IgG for mouse primary antibodies, or antibody diluent for rabbit antibodies. Each wash was done in 4ml of PBS; cells were collected by centrifuging at 320g for 5 min. If cell-permeabilization was needed (for primary antibodies recognising intracellular epitopes), saponin (0.05%, final concentration) was added to the antibody diluent and washing buffer. After incubation with primary antibody, the cells were washed twice, then incubated with 10 µg/ml of Alexa Fluor® 488 conjugated secondary antibodies for 45 min. Cells were washed twice, suspended in PBS, then analysed by FACSCalibur™ using Cell Quest software (Becton Dickinson, UK). For each sample, data from 10,000 cells were collected. Each determination was performed with 3 or 4 replicates and the data is expressed as the mean (±SEM) of the median fluorescence from each replicate. Experiments compared different conditions using paired t-tests.

### Transmission electron microscopy (TEM)

3D co-culture gels prepared on transwell inserts were washed 3× in PBS and fixed in 2.5% glutaraldehyde in Sörensons phosphate buffer (PB) for 1 hour. Post-fixation was carried out with 1% (w/v) osmium tetroxide in 0.1M PB for 1 hour and the inserts were then washed in 0.1M PB for 10 min. The filters were excised from the insert and randomly cut into 2 segments of 3–5mm × 2mm. These segments were progressively dehydrated in a graded series of ethanol from 30% to 100%, embedded in Epon resin (Agar Scientific, UK) and polymerised at 60°C for 48h. Ultrathin sectioning was performed using a Diatome diamond knife on a Leica Ultracut UCT (Leica Biosystems Inc, UK), producing sections of 80–90nm thickness, which were then collected on 2×1mm copper grids coated with pioloform film. The sections were counterstained at room temperature with 4% aqueous uranyl acetate for 35 min, washed 3×, immersed in Reynolds lead citrate, (pH 12), for 10 min in a CO _2_-free environment, and finally washed 3× before air-drying. The sections were observed on a transmission electron microscope JEM-1400 (Jeol, Japan) operated at an accelerating voltage of 80 kV using a magnification of ×5000 to ×25000. In experiments with gold nanoparticles, silver enhancement (R-Gent SE-EM, Aurion, Netherlands) was carried out for 45 min at room temperature, according to the manufacturer’s protocol, before processing as described above.

### Data analyses

Fluorescence intensity analysis was carried out using ImagePro software and the percentage of pixels exceeding a threshold pixel-density was measured, with a threshold set at mean+2SD of background. Flow cytometry was carried out using FACSCalibur™ to analyse 10 ^4^ cells on each determination, using CellQuest software (Becton Dickinson, UK), to give median fluorescence. The gain voltage was set so that 90% of control, stained cells had fluorescence values <10. Cells with fluorescence >10 were considered positive. Graphical representations and statistical analyses were done with Graphpad Prism software, v. 3.0 (Prism, USA). Data were analysed by Anova, followed (if *P*< 0.05) by either Dunnet’s multiple comparison test, a t-test or a paired t-test as appropriate.

## Results

### Phenotype of human astrocytes in 3D gels

The characteristics of astrocytes cultured in collagen gels (3D-astrocytes) for 7 days were compared to the same cells cultured on flasks (2D-astrocytes). Cells grown on flasks had increased in number approximately 7-fold over this period, equivalent to 2.9 ± 0.08 cell divisions. In contrast, there was no significant increase in the numbers of cells present in the gels after 7 days in culture. The difference between 2D-astrocytes and 3D-astrocytes ( *P*=0.0009, paired t-test, n=4) indicates that culture in the 3D gel environment inhibits astrocyte proliferation. Over the same time period, the percentage of TE7 ^+^ fibroblasts increased significantly from 2% to 5% in both 3D-cultures ( *P*=0.0483, paired t-test, n=3) and 2D cultures ( *P*=0.0428, paired t-test, n=3), indicating that fibroblasts can proliferate slowly in the gels. Representative images of TE7 fibroblast staining are shown in Supplementary Figure 1.

 Over this period the percentage of GFAP ^+^ cells was unchanged in the 3D gels, while the percentage in monolayer cultures fell from 39% to 20% ( *P*=0.0309). The maintenance of GFAP expression in 3D cultures of human astrocytes contrasts with previous results with rat astrocytes, which down-regulate GFAP expression in gel cultures ( East *et al.*, 2009). While 90% of the 2D-astrocytes expressed S100β, only approximately half of them were GFAP ^+^. GFAP is often used as a standard marker of astrocytes, but in this study S100β appeared to be more consistently expressed in human astrocytes under different growth conditions than GFAP. Images of GFAP staining on astrocytes in 2D and 3D cultures are shown in Supplementary Figure 2.

Raw data for the statements made in the Results ‘Phenotype of human astrocytes in 3D gels’ Percentage of TE7- and GFAP- positive cells in Human astrocyte 2D and 3D cultures after 7 days ( Sreekanthreddy *et al.*, 2015a). Click here for additional data file. Copyright: © 2016 Sreekanthreddy P et al. 2016 Data associated with the article are available under the terms of the Creative Commons Zero "No rights reserved" data waiver (CC0 1.0 Public domain dedication).

### Astrocyte-endothelial co-cultures

**Figure 1.**
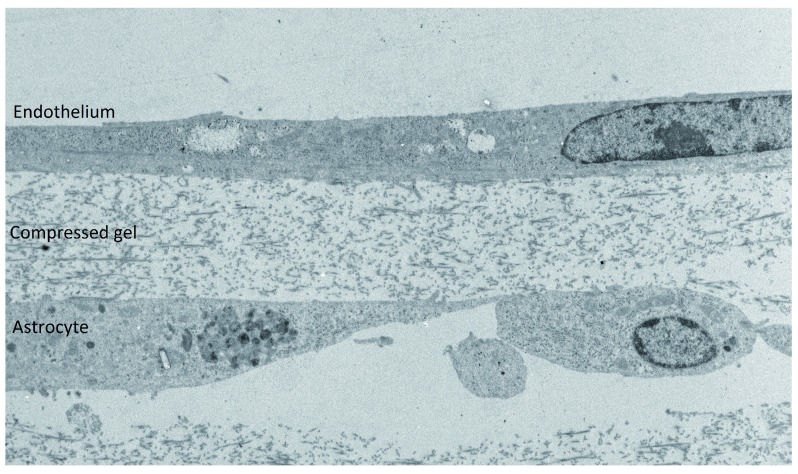
3-dimensional brain endothelium/astrocyte co-culture. Electron micrograph of a transverse section (85nm) of a compressed collagen type-1 gel with a co-culture of 3D-astrocytes and hCMEC/D3 endothelial cells; astrocytes occupy 6–10% of the gel as measured by image area. Scale bar = 2μm.

In the next step, hCMEC/D3 cells were added to the surface of compressed or uncompressed gels containing astrocytes ( Figure 1). Compression increased the density of astrocytes and also made the gels considerably more resilient for TEM processing. In order to estimate the volume occupied by the astrocytes in the gels, the total area of the astrocytes was measured as a percentage of the gel, by image analysis of strips across the width of the cultures. In uncompressed gels (1–2mm thick) the astrocytes occupied <1% of the gel, and in compressed gels (40–60μm thick) they occupied 6–10% of the gel ( Figure 1). Direct contact between astrocytes at the surface of the gel and endothelium could be seen in TEM ( Supplementary Figure 3). However, since the astrocyte density was always <10% of the gel volume the contact area between astrocytes and endothelium was less than 10% of the endothelial monolayer and the permeability of the co-cultures to a 70kDa dextran tracer was not significantly different to monocultures of hCMEC/D3 cells on transwell inserts ( *P>*0.05, t-test, n=3). The upper limit on astrocyte density in the 3D cultures was determined by gel contraction and higher densities of astrocytes could be tolerated in compressed gels, due to their greater rigidity. The model allows analysis of local contacts between the astrocytes and endothelium by fluorescence or electron microscopy, which is an advantage over conventional co-cultures where the endothelium and astrocytes are separated by a filter up to 30µm thick, and direct contact has not been observed.

### Cell phenotypes in co-culture

**Figure 2.**
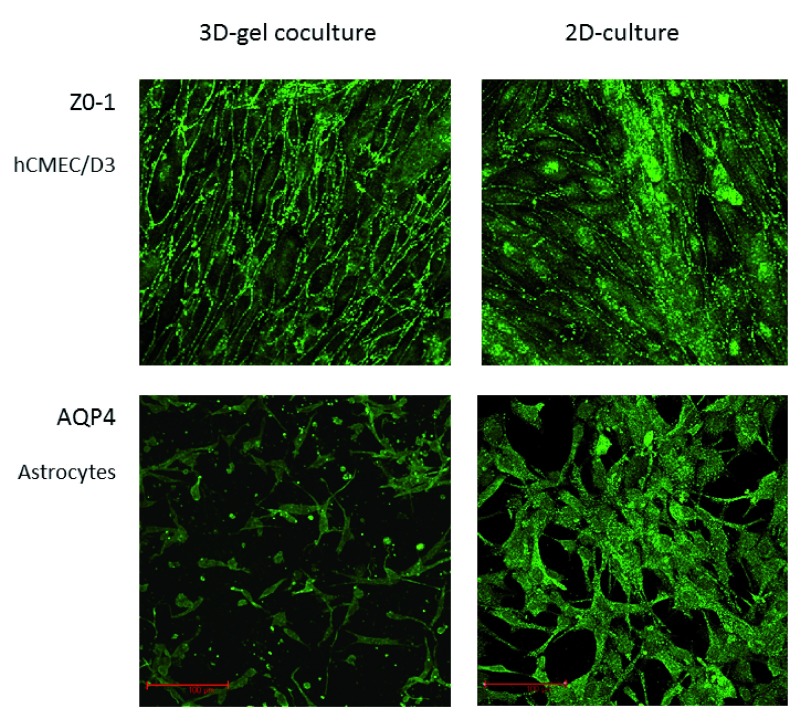
Expression of ZO-1 and AQP4 in 2D- and 3D-astrocyte cultures. Immunofluorescence staining *in situ* for expression of ZO-1 in hCMEC/D3 cells (top) and AQP4 in human primary astrocytes (bottom) in 3D gel co-cultures (left) compared with conventional 2D solo cultures on a hard surface (right). 2D and 3D cultures were prepared, cultured, stained and imaged under the same conditions.

Another advantage of the gel-based 3D co-culture is that either cell type can be readily observed by microscopy: the transparency of the collagen matrix allows imaging of the endothelial monolayer and astrocytes at different depths in the gel, by conventional light or fluorescence confocal microscopy. Immunofluorescence microscopy was therefore used to examine the expression of characteristic molecules on the endothelium or the astrocytes in the 3D co-cultures. To assess expression of tight junction molecules, endothelial cells were immunostained with anti-ZO-1 and anti-CLDN5. Staining for ZO-1 was continuous around the edge of the cells ( Figure 2, upper), whereas CLDN5 was irregular, as described previously ( Weksler *et al.*, 2005) in both 2D and 3D cultures. Immunofluorescence staining of the tight junctions on the endothelium indicated that the structural organisation of the junctions was similar to that seen in conventional solo cultures of hCMEC/D3 cells, grown on collagen-coated tissue-culture flasks. Staining of the astrocytes for AQP4 ( Figure 2, lower) produced a homogeneous surface staining, with apparently lower expression on astrocytes in 3D co-cultures.

**Figure 3.**
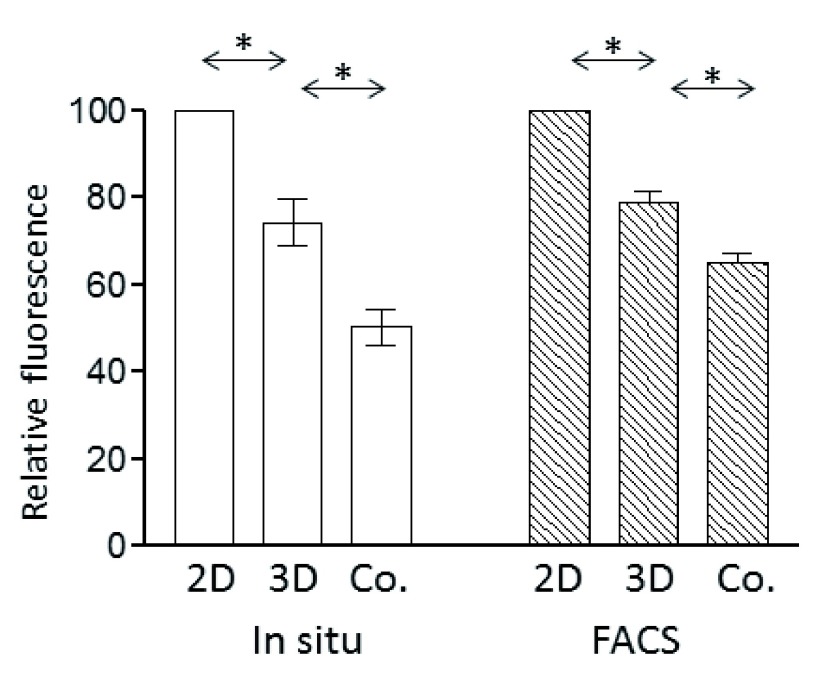
Modulation of astrocyte AQP4 in 3D barrier model. Expression of AQP4 in 2D-astrocytes, 3D-astrocytes or astrocytes in 3D co-cultures (Co.) measured by immunofluorescence *in situ* and image analysis (left), or by isolation of the cells and flow cytometry (right). Fluorescence is expressed relative to the median value in 2D-astrocytes. Data points are the mean (±SEM) of median fluorescence values. 3D-astrocytes expressed lower levels of AQP4 than 2D-astrocytes and the expression of 3D-astrocytes further decreased in co-cultures (* P<0.05, paired t-test, n=3).

Two previous studies indicated that contact with endothelium can affect expression and localisation of aquaporin-4 (AQP4) in astrocytes ( Al Ahmad *et al.*, 2011; Nicchia *et al.*, 2004). To investigate whether co-culture with endothelium was indeed affecting AQP4, we compared expression in 2D-astrocytes, 3D-astrocyte cultures and co-cultures. Culture of astrocytes in 3D gels, by itself causes a reduction in AQP4 expression both by immunofluorescence *in situ* (by image analysis) and by flow cytometry ( Figure 3). The expression was further reduced when the astrocytes were in co-culture with endothelium ( Figure 3). We did not observe any structural reorganisation of AQP4 to the contact points between the endothelium and astrocytes in these co-cultures, but as the overall area of contact was limited, immunofluorescence is not really suitable for detecting such highly localised changes. Expression of the endothelin receptor, type B (EDNRB) was also reduced by approximately 16%, on 3D-astrocytes by comparison with 2D-astrocytes as measured by flow cytometry (paired t-test, *P*=0.028 n=3). However, there was no additional change when the 3D-astrocytes were in a co-culture with endothelium.

Raw data for aquaporin-4 expression (Figure 3) AQP4 levels tested through flow cytometry and *in-situ* gel imaging analysis with results from paired t-tests ( Sreekanthreddy *et al.*, 2015b). Click here for additional data file. Copyright: © 2016 Sreekanthreddy P et al. 2016 Data associated with the article are available under the terms of the Creative Commons Zero "No rights reserved" data waiver (CC0 1.0 Public domain dedication).

**Figure 4.**
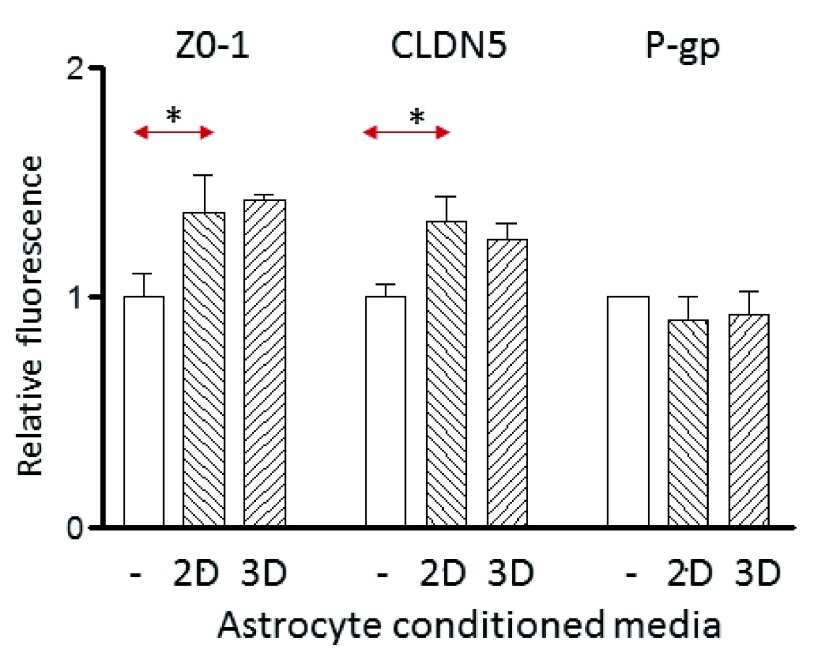
Modulation of ZO-1 and CLDN5 in 3D barrier model. Expression of ZO-1, CLDN5 and P-glycoprotein (P-gp) on hCMEC/D3 cells cultured in control medium from endothelium (-), or 50% astrocyte-conditioned medium from either 2D- or 3D-astrocytes. The values obtained by fluorescence microscopy are expressed relative to the median fluorescence of control-treated cells in 3 independent experiments. Data points are the mean (±SEM) of the median fluorescence. ZO-1 and CLDN5 are induced by 2D-astrocyte conditioned medium (* P<0.05, paired t-test, n=3), but there was no significant difference between 2D-astrocytes and 3D-astrocytes.

It has previously been shown that astrocyte-conditioned medium (ACM) can enhance barrier properties in brain endothelium ( Siddharthan *et al.*, 2007) and induce the multi-drug transporter, P-glycoprotein ( Megard *et al.*, 2002). We therefore examined whether the factors released from 3D-astrocytes were more or less effective in inducing barrier phenotype in endothelial cells than those from 2D-astrocytes. hCMEC/D3 cells were cultured with 50% conditioned medium from either 2D-astrocytes or 3D-astrocytes and the area of staining measured by image analysis. The ACM from both sources induced ZO-1 and CLDN5 expression in hCMEC/D3 cells to a similar degree ( Figure 4), but neither had any effect on P-glycoprotein expression ( Figure 4).

Raw data for expression of ZO-1, CLDN5 and Pgp (Figure 4) Raw data for ZO-1 expression: Z0-1 expression-Fig-4.xlsx; Raw data for CLDN5 expression: CLDN5 expression-Fig-4.xlsx; Raw data for Pgp-1 expression: Pgp-1 expression-Fig-4.xls. Files show expression levels with basic statistics (mean, stdev, SEM) and paired t-test results ( Sreekanthreddy *et al.*, 2015c). Click here for additional data file. Copyright: © 2016 Sreekanthreddy P et al. 2016 Data associated with the article are available under the terms of the Creative Commons Zero "No rights reserved" data waiver (CC0 1.0 Public domain dedication).

### Use of the model for nanoparticle transfer and leukocyte migration

**Figure 5.**
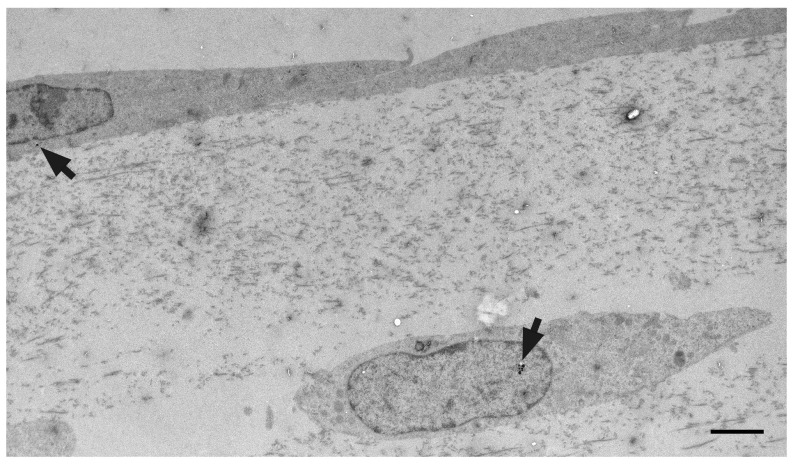
Co-culture model used to assess nanoparticle transmigration. Electron micrograph of a 3D co-culture of human primary astrocytes and hCMEC/D3 cells after application of 8μg/ml glucose-coated gold nanoparticles to the endothelial surface. Nanoparticles (arrows) were detected both in the endothelium and the astrocytes. Scale bar = 500nm.

To examine the use of the model for assessing nanoparticle transport, 3D co-cultures were set up on transwell inserts on 24-well plates and overlaid with 8μg/ml of 4nm glucose-coated gold nanoparticles ( Gromnicova *et al.*, 2013). At intervals of 1–22 hours the cultures were fixed and examined for the localisation of nanoparticles by TEM following silver enhancement ( Figure 5, Supplementary Figure 4). The micrographs showed that these nanoparticles were located in the endothelium, the gel matrix and at later time points in astrocytes, suggesting that they move across the endothelium, and then diffuse through the gel matrix and then enter astrocytes (this class of nanoparticle is known to be able to cross plasma membranes directly ( Verma *et al.*, 2008)). The model allows quantitation of the numbers of nanoparticles in the endothelium and astrocytes by particle counting, and it allows study of subcellular localisation of the nanoparticles. The model has recently been used for this purpose ( Gromnicova *et al.*, 2013). It is also theoretically possible to quantitate cell-associated and matrix-associated gold nanoparticles (by mass spectrometry) following separation of the components of the co-culture by the 2-step collagenase method.

**Figure 6.**
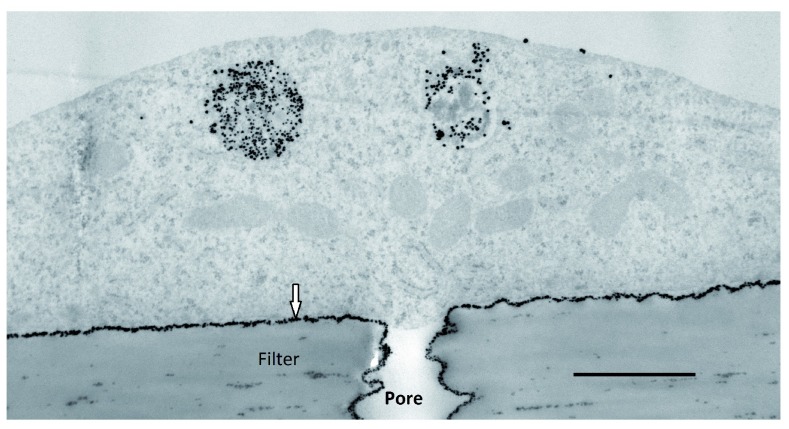
Limitations of transwells for assessing nanoparticle transmigration. Electron micrograph of a section through hCMEC/D3 cells growing on a transwell filter, 1 hr after application of insulin-coated 4nm gold nanoparticles to the apical surface. The cells were fixed and nanoparticles visualised by silver enhancement. Nanoparticles (arrow) are trapped between the filter and basal membrane, except where there are pores (pore) in the filter. Scale bar = 1µm.

One particular advantage of the present 3D gel co-culture is that it allows nanoparticles to be released from the entire basal membrane of the endothelium. In contrast, if endothelium is cultured directly on top of transwell filters, nanoparticles become trapped between the basal plasma membrane and the filter, except where there are pores ( Figure 6). Moreover some types of nanoparticle bind strongly to filters and for these nanoparticles transwells are not suitable for quantitation of transfer rates, because of the losses on the filter.

**Figure 7.**
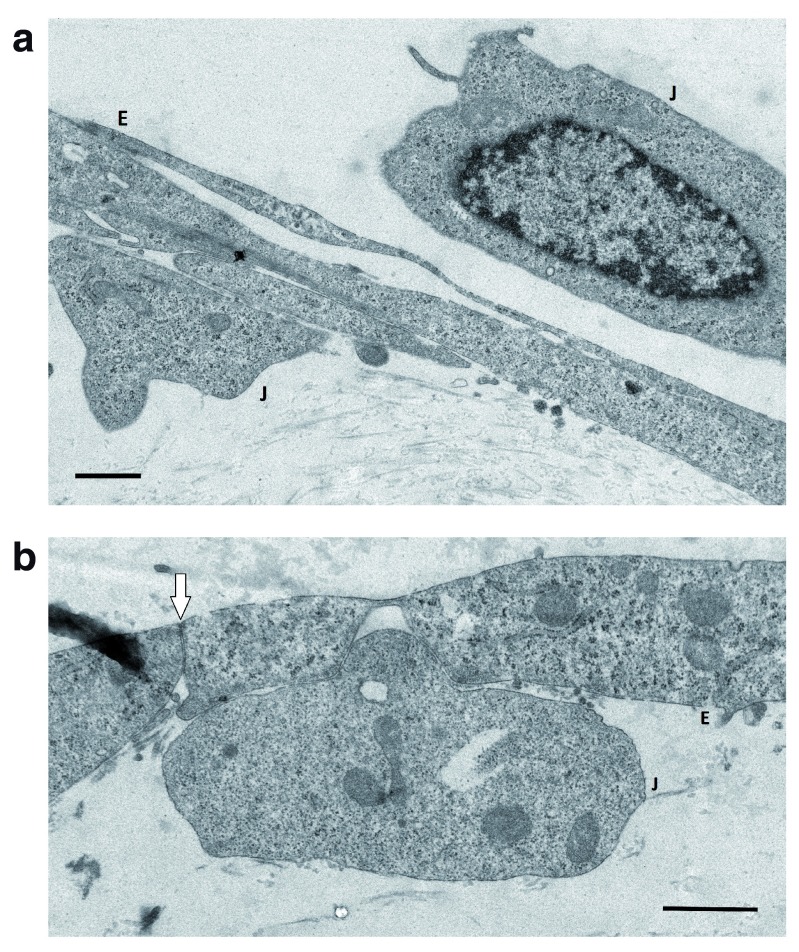
Co-culture model used to measure leukocyte transmigration. Migration of Jurkat cells in a 3D-astrocyte-endothelial co-culture. **a**) At 3h Jurkat cells (J) are seen above and below the endothelial cells (E), with some cellular processes interleaved in the endothelial monolayer. **b**) At 24h the Jurkat cells are located between the endothelium and the collagen gel. The arrow indicates an intact inter-endothelial junction. Transendothelial migration occurs by emperiopolesis. Scale bar = 1μm.

We also examined the model to determine whether it could be used to assess leukocyte migration across the endothelium and through the gel. Collagen hydrogel co-cultures were established in transwell inserts and the endothelium and astrocytes were activated by treatment with 1–10ng/ml TNFα plus IFNγ, either in the upper or lower chambers. Earlier studies have established that these conditions induce appropriate adhesion molecules and chemokine synthesis by the endothelium ( Subileau *et al.*, 2009). The endothelium was then overlaid with 10 ^5^ Jurkat cells labelled with CMFDA and incubated for 16–24h. At the end of the incubation the cultures were observed by fluorescence microscopy and TEM. Confocal microscopy identified fluorescent lymphocytes associated with the endothelial monolayer, and with smaller numbers up to 300μm deep in the gel ( Supplementary Figure 5). Migration into the gel was only seen with uncompressed gels. The TEM pictures demonstrated that the lymphocytes had migrated through the endothelium, exhibiting the classical appearance of emperipolesis ( Figure 7). The micrographs indicated that the majority of the leukocytes detected had accumulated below the basal plasma membrane of the endothelium but had not migrated into the gel at 24h ( Supplementary Figure 6). Accumulation of leukocytes below the plasma membrane of brain endothelium but within the glia limitans (perivascular cuffing) is also a characteristic of leukocyte migration into the CNS *in vivo*.

## Discussion

The 3D co-culture model was developed to assess interactions between human brain endothelium and astrocytes and for its potential to measure trans-endothelial migration of nanoparticles and leukocytes. The configuration of these cultures offers three potential advantages: firstly astrocytes can directly contact the endothelium; secondly, compared to 2D hard surface cultures, the 3D soft collagen gel matrix provides a physiologically more realistic cell environment, as indicated by inhibition of astrocyte proliferation; thirdly, nanoparticles, cells or biological agents released from the basal plasma membrane of the endothelium can directly enter the gel and/or astrocytes.

The model also has great potential for investigating the phenotype of the two cell types in co-culture, by light and fluorescence microscopy due to the transparency of the matrix. Compressed gels are also readily processed for TEM. Moreover as both cell types can be recovered separately by collagenase digestion, they can be individually analysed by many other techniques.

The second major use of this model was intended to be in measuring the movement of nanoparticles, across brain endothelium and into astrocytes. Delivery of therapeutic transgenes to cells within the CNS holds great potential for treatment of CNS diseases ( Manfredson & Mandel, 2010), but developing systems that deliver the transgene to the target cell within the CNS is a challenge. Nanoparticles have the capacity to act both as drug carriers and transporters of therapeutic transgenes ( Kanwar *et al.*, 2012). However there is currently no convenient system for measuring trans-endothelial movement of the nanoparticles *in vitro* and localising them in target cells. This model is well suited for this purpose. Nanoparticles can be tracked and quantitated by TEM (or light microscopy for larger particles). We have now used this model to quantitate delivery of different classes of nanoparticle to astrocytes in the gels and determine the rate and route of transfer across the endothelium ( Gromnicova *et al.*, 2013). We also evaluated the model for leukocyte migration. In comparison with another recently-developed astrocyte/endothelial co-culture ( Takeshita *et al.*, 2014), specifically designed for measuring leukocyte migration, our model lacks shear stress, and is less suited for quantitation of leukocyte traffic, but can be used for characterising the trans-migrating cells and identifying their routes of migration.

Another related model which includes rat brain endothelium (RBE4-line), rat primary astrocytes and primary pericytes, in a collagen gel has been developed previously ( Al Ahmad *et al.*, 2011). In this case all cells were incorporated into the gel matrix as the model was designed to investigate angiogenesis, and tubulogenesis. In comparison, it is less well suited for the analysis of mechanisms of transcytosis.

## Conclusions

This 3D co-culture system with quiescent astrocytes in a collagen hydrogel overlaid with a monolayer of brain endothelium can be used for examining astrocyte-endothelial interactions; the two cell types can directly contact each other, although the overall contact area is limited. With only two cell types, the model is relatively easy to prepare in quantity. The model can also be used for investigating leukocyte migration across brain endothelium. It is particularly well suited for measuring the delivery of nanoparticles, transgenes and larger biological molecules to cells of the CNS, following transfer across the endothelium, because these larger entities can move freely from the basal surface of the endothelium, through the gel matrix and into the target cells. The co-culture is less suitable for modelling movement of small molecules across brain endothelium.

## Data availability

The data referenced by this article are under copyright with the following copyright statement: Copyright: © 2016 Sreekanthreddy P et al.

Data associated with the article are available under the terms of the Creative Commons Zero "No rights reserved" data waiver (CC0 1.0 Public domain dedication). 

*F1000Research*: Dataset 1. Raw data for the statements made in the Results ‘Phenotype of human astrocytes in 3D gels’, 10.5256/f1000research.7142.d106539

*F1000Research*: Dataset 2. Raw data for aquaporin-4 expression ( Figure 3), 10.5256/f1000research.7142.d106541

*F1000Research*: Dataset 3. Raw data for expression of ZO-1, CLDN5 and Pgp ( Figure 4), 10.5256/f1000research.7142.d106543

## Supplementary material

**Supplementary Figures 1–6.**

**Supplementary Figure 1**: TE7 staining of fibroblasts in astrocyte cultures. Green staining indicates TE7 expression. Blue staining by DAPI indicates the nucleus of the cell. Representative images of each culture condition are shown here. **A**: Day-1 2D culture. **B**: Day-8 2D culture. **C**: Day-8 3D culture. Scale bars represents 200 µm. **D**: Double staining for TE7 (red) and GFAP (green) on 2D astrocyte culture at passage 8. Contaminating fibroblasts (TE7+) are distinct from GFAP+ astrocytes.

**Supplementary Figure 2**: Green fibrillary, cytoplasmic staining indicates GFAP expression. Blue staining by DAPI indicates the nucleus of the cell. Representative images of each culture condition are shown here. **A**: Day-1 2D culture. **B**: Day-8 2D culture. **C**: Day-8 3D culture. Scale bar represents 200 µm.

**Supplementary Figure 3**: Electron micrograph of a 3D-astrocyte endothelial cell co-culture (uncompressed). Astrocytes constitute approximately 1% of the gel volume and in some places are in direct contact with the basal plasma membrane of the hCMEC/D3 cells. Scale bar = 2mm.

**Supplementary Figure 4**: Electron micrograph of hCMEC/D3 cell 8 hrs after application of glucose-coated 4nm gold nanoparticles to the apical surface. The cells were fixed and nanoparticles visualised by silver enhancement. Nanoparticles are present in the cytosol and below the basal membrane (arrows). Scale bar = 500nm.

**Supplementary Figure 5**: Confocal micrograph of CMFDA-labelled Jurkat cells migrating into a 3D uncompressed co-culture. The endothelium was stimulated with 1ng/ml TNFα and 1ng/ml IFNγ at time 0. Jurkat cells were applied to the apical surface and allowed to migrate for 24h before fixation. Nuclei of the endothelial cells are labelled with DAPI. Some Jurkat cells are associated with the endothelial monolayer, while others have migrated up to 300mm into the gel. Scale bar = 200mm.

**Supplementary Figure 6**: Electron micrograph of Jurkat cells (J) migrating into a 3D uncompressed co-culture. Migration into the gel occurs at a point where the endothelial monolayer (E) is incomplete. Some astrocytes (A) are also visible in this section. Scale bar = 1mm.
