# Supplementary material for: A three-dimensional model of the human blood-brain barrier to analyse the transport of nanoparticles and astrocyte/endothelial interactions
Source: F1000Res. 2016 Jan 21;4:1279. Originally published 2015 Nov 17. [Version 2] doi: 10.12688/f1000research.7142.2 (PMC4732555; doi:10.12688/f1000research.7142.2)
Supplement: Supplementary file 4 [file f1000research-4-8385-s0003.tgz › ae0d871a-2d00-4164-a3d0-3dbbf5f37c80.pptx]

## Slide 1
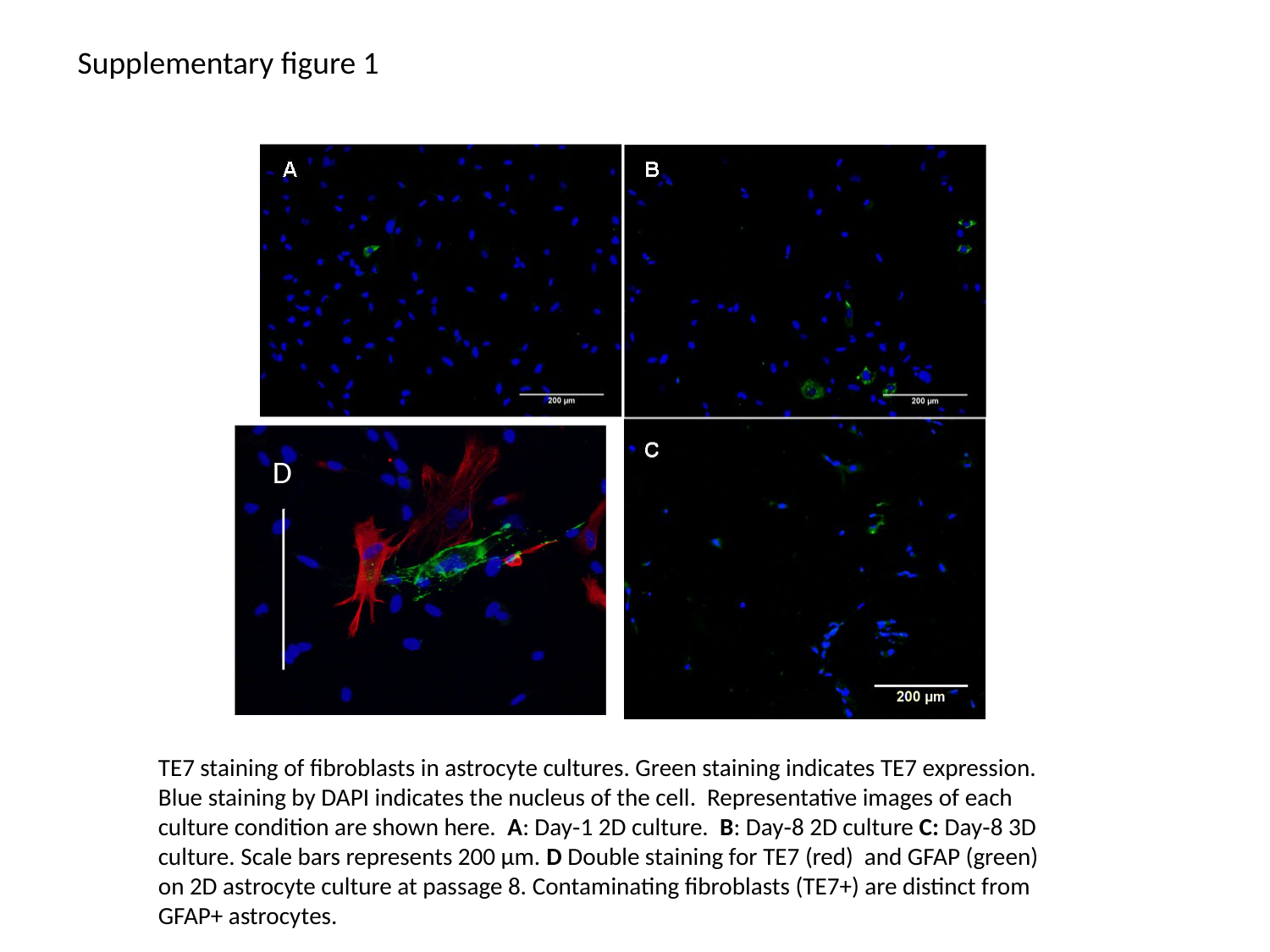

Supplementary figure 1
D
TE7 staining of fibroblasts in astrocyte cultures. Green staining indicates TE7 expression. Blue staining by DAPI indicates the nucleus of the cell. Representative images of each culture condition are shown here. A: Day‑1 2D culture. B: Day‑8 2D culture C: Day‑8 3D culture. Scale bars represents 200 µm. D Double staining for TE7 (red) and GFAP (green) on 2D astrocyte culture at passage 8. Contaminating fibroblasts (TE7+) are distinct from GFAP+ astrocytes.

## Slide 2
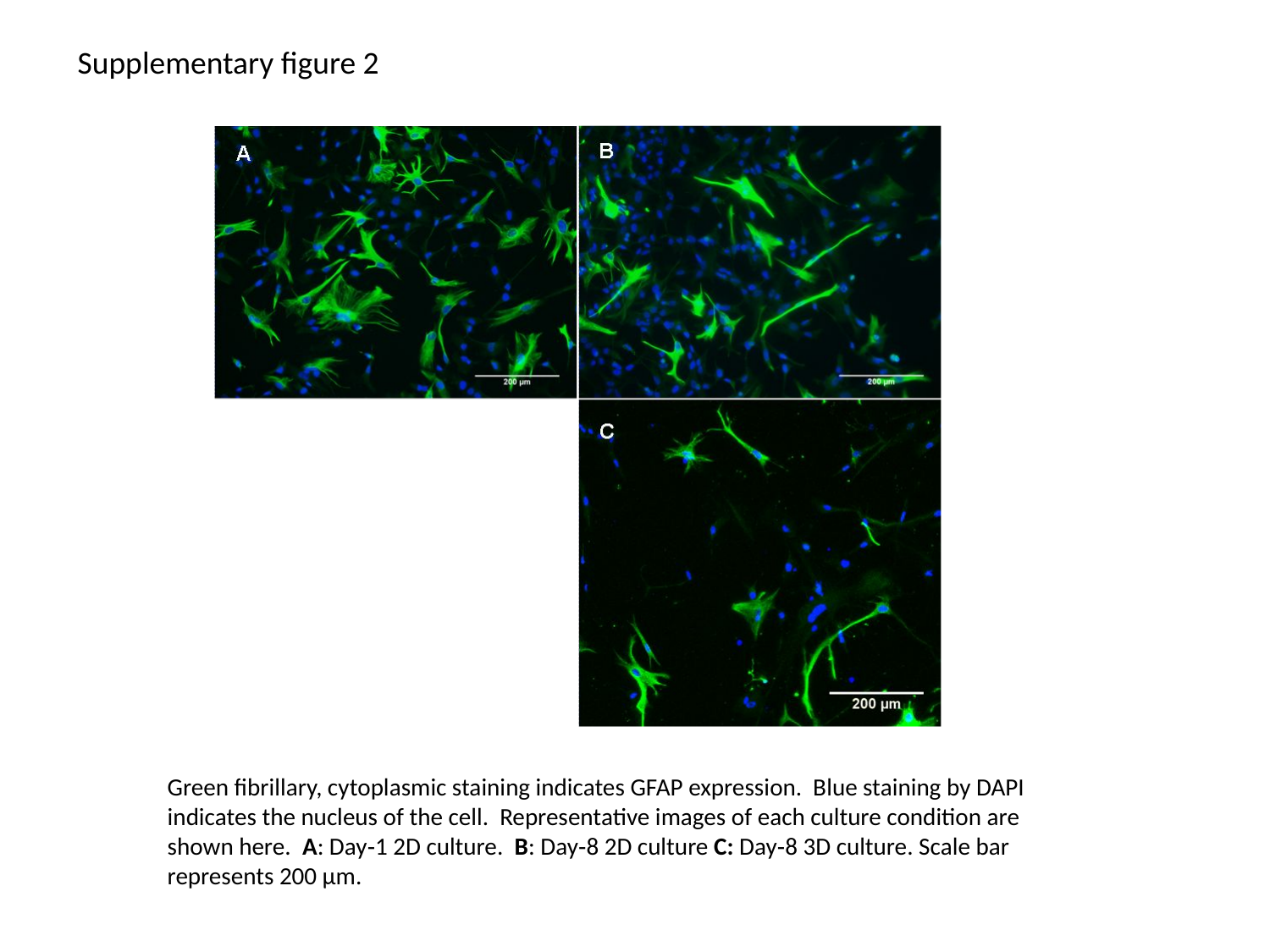

Supplementary figure 2
Green fibrillary, cytoplasmic staining indicates GFAP expression. Blue staining by DAPI indicates the nucleus of the cell. Representative images of each culture condition are shown here. A: Day‑1 2D culture. B: Day‑8 2D culture C: Day‑8 3D culture. Scale bar represents 200 µm.

## Slide 3
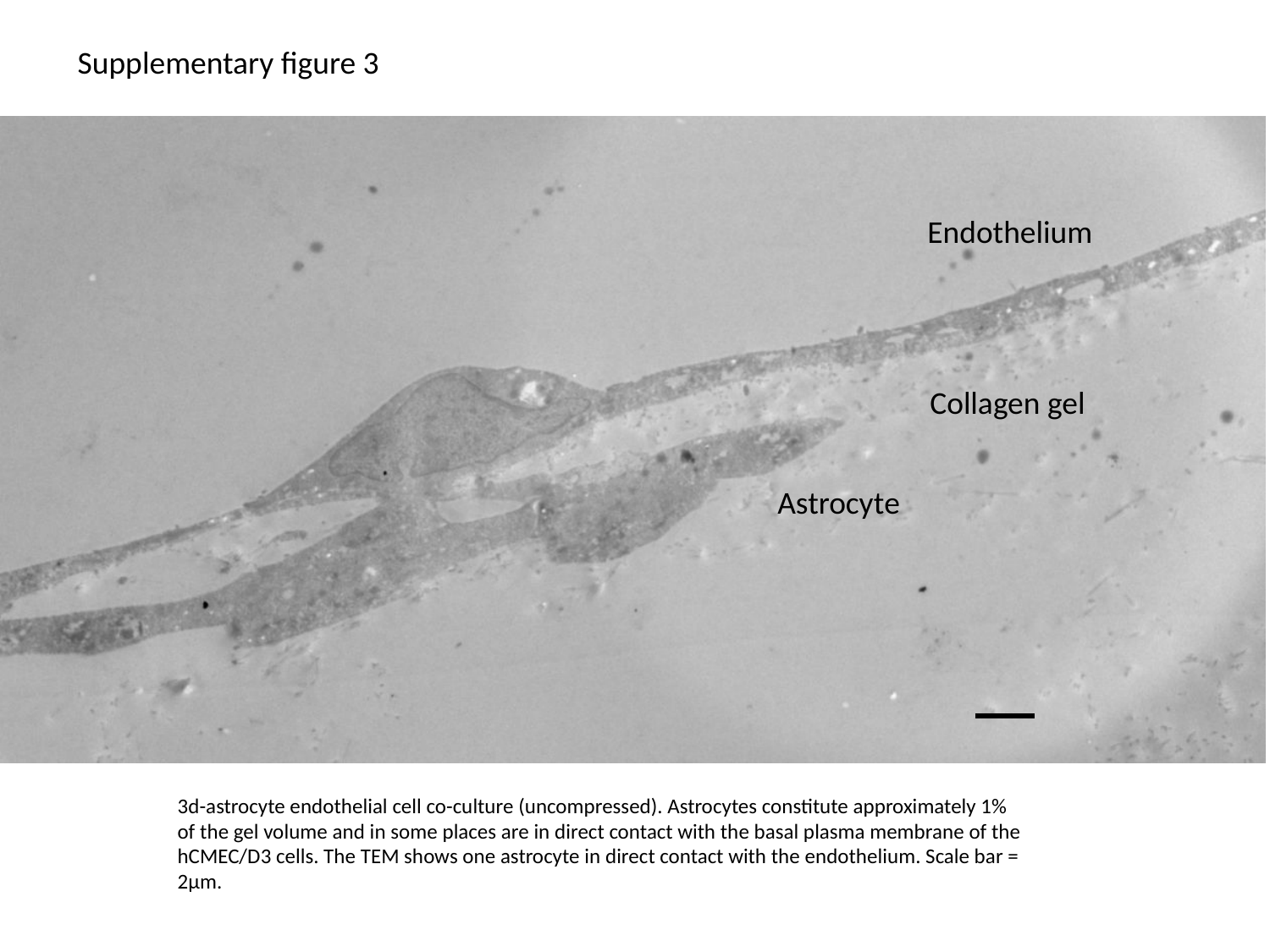

Supplementary figure 3
Endothelium
Collagen gel
Astrocyte
3d-astrocyte endothelial cell co-culture (uncompressed). Astrocytes constitute approximately 1% of the gel volume and in some places are in direct contact with the basal plasma membrane of the hCMEC/D3 cells. The TEM shows one astrocyte in direct contact with the endothelium. Scale bar = 2μm.

## Slide 4
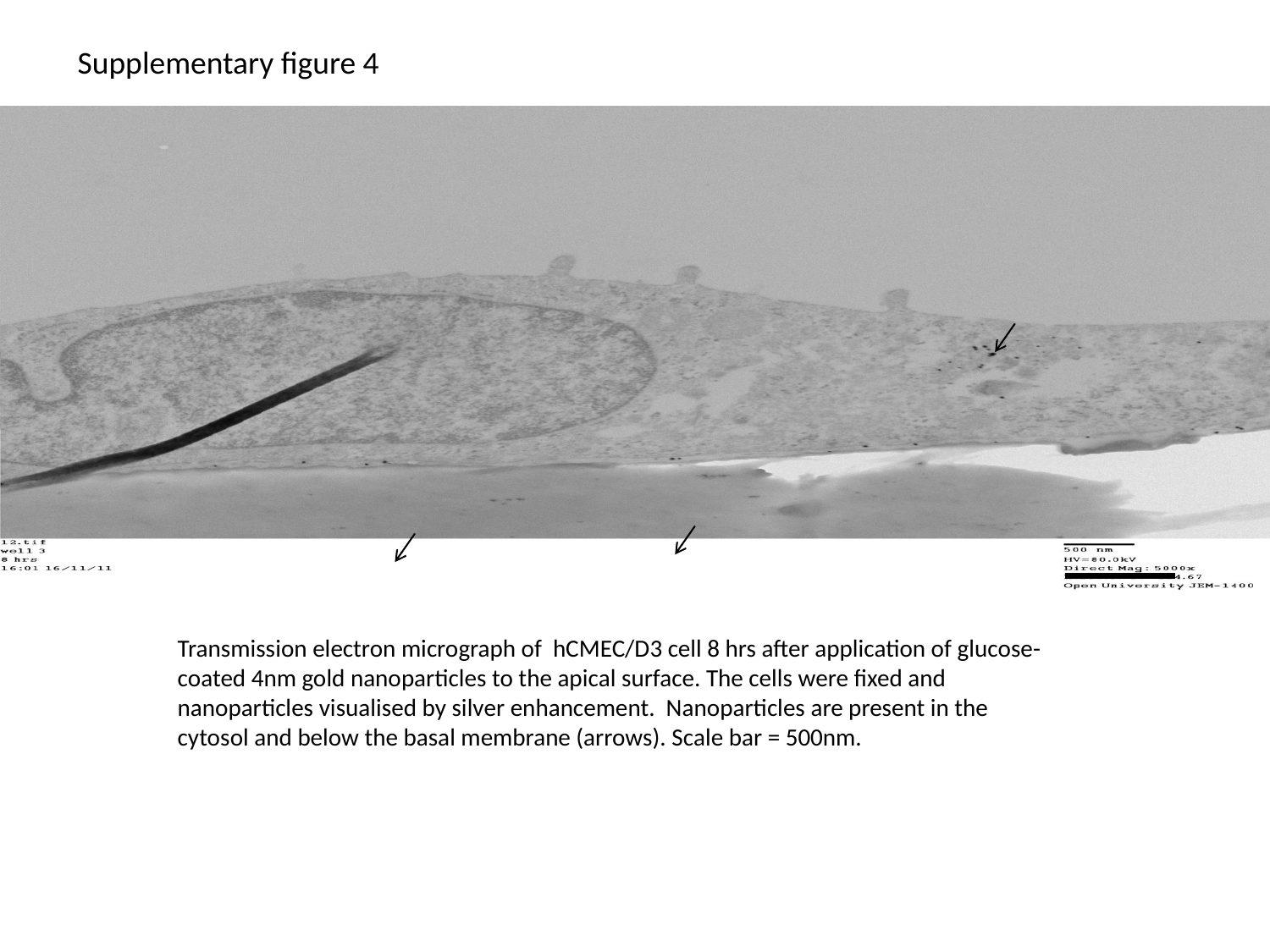

Supplementary figure 4
Transmission electron micrograph of hCMEC/D3 cell 8 hrs after application of glucose-coated 4nm gold nanoparticles to the apical surface. The cells were fixed and nanoparticles visualised by silver enhancement. Nanoparticles are present in the cytosol and below the basal membrane (arrows). Scale bar = 500nm.

## Slide 5
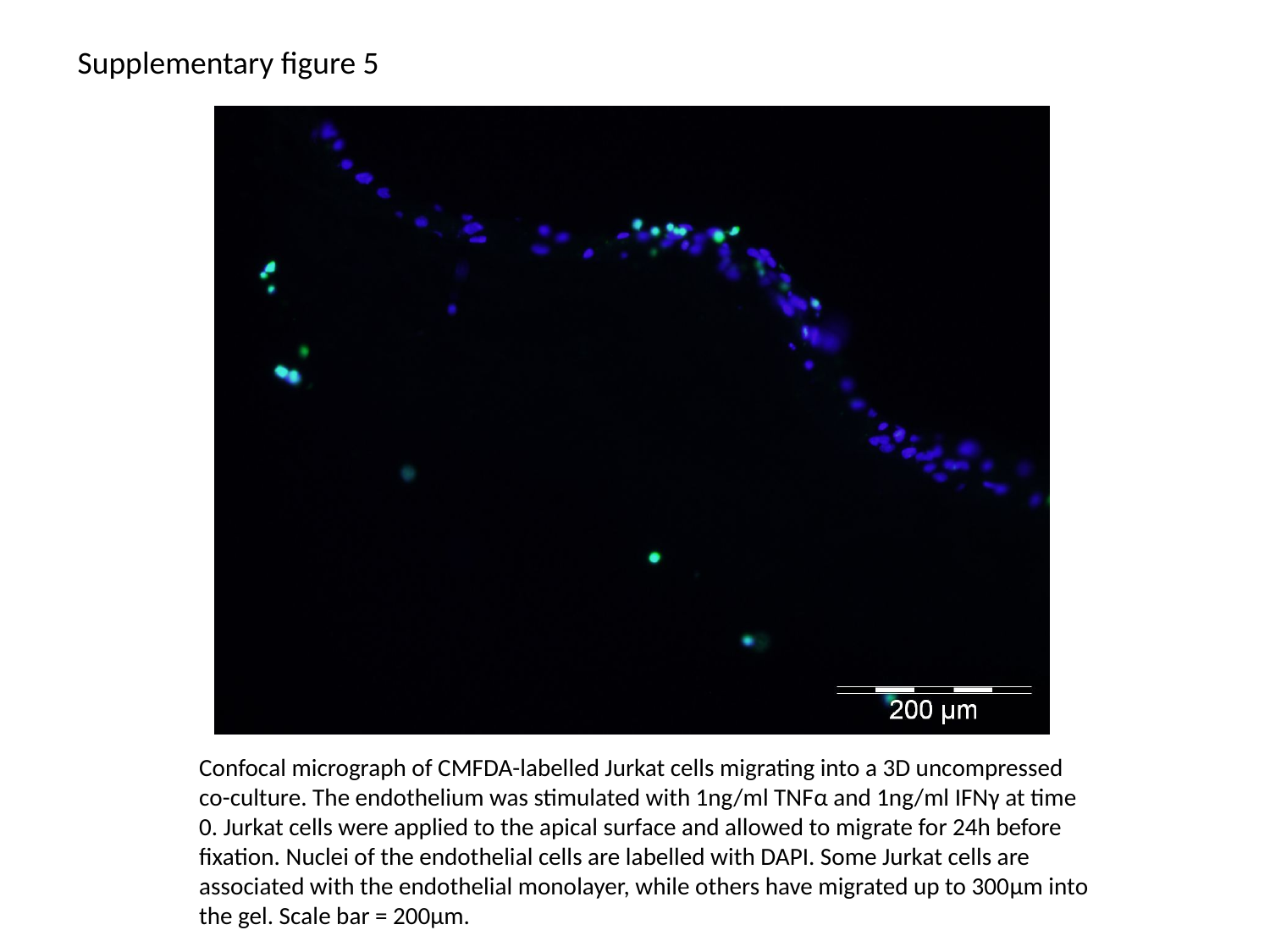

Supplementary figure 5
Confocal micrograph of CMFDA-labelled Jurkat cells migrating into a 3D uncompressed co-culture. The endothelium was stimulated with 1ng/ml TNFα and 1ng/ml IFNγ at time 0. Jurkat cells were applied to the apical surface and allowed to migrate for 24h before fixation. Nuclei of the endothelial cells are labelled with DAPI. Some Jurkat cells are associated with the endothelial monolayer, while others have migrated up to 300μm into the gel. Scale bar = 200µm.

## Slide 6
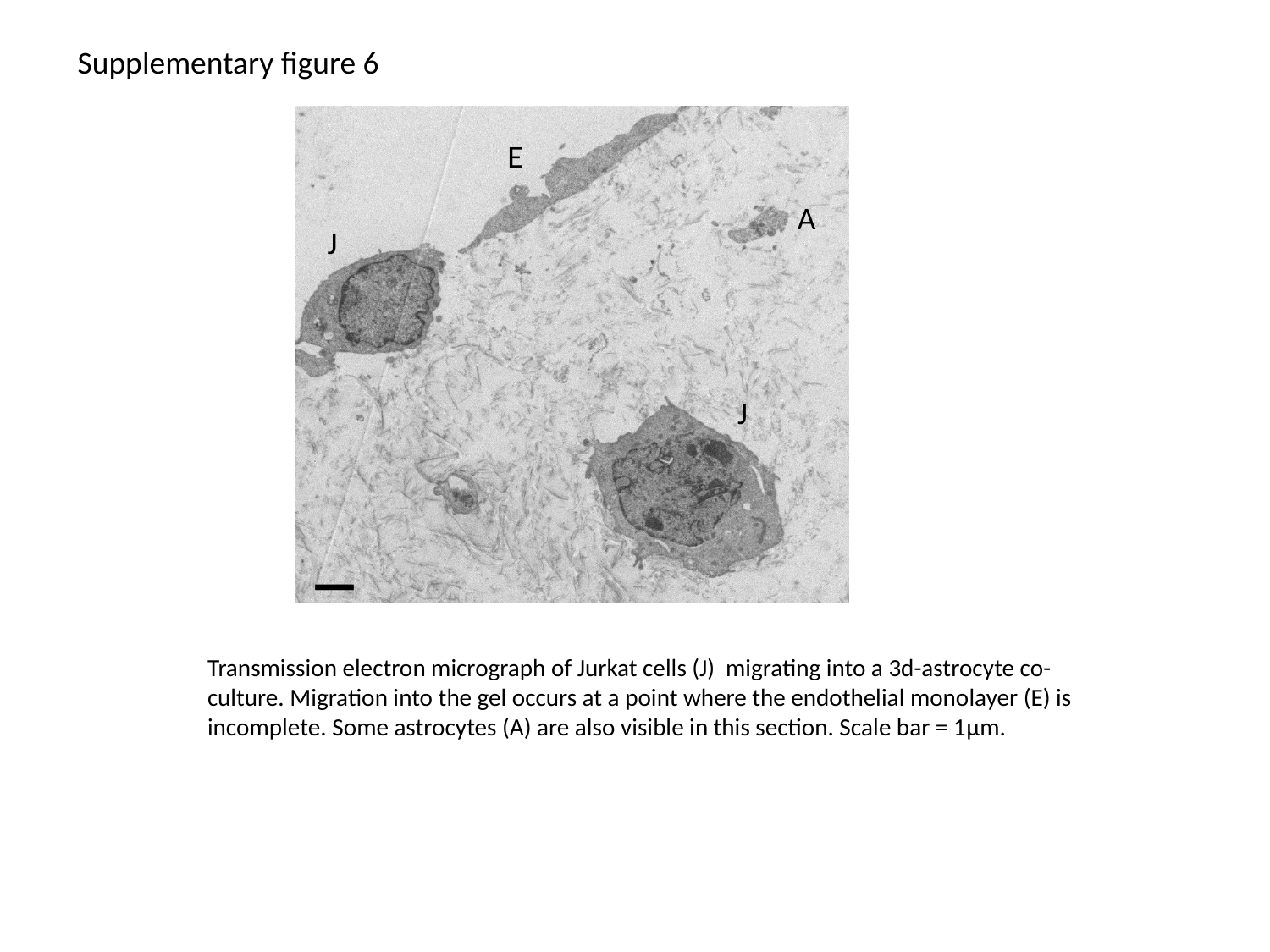

Supplementary figure 6
E
A
J
J
Transmission electron micrograph of Jurkat cells (J) migrating into a 3d-astrocyte co-culture. Migration into the gel occurs at a point where the endothelial monolayer (E) is incomplete. Some astrocytes (A) are also visible in this section. Scale bar = 1μm.
