# Supplementary material for: A three-dimensional model of the human blood-brain barrier to analyse the transport of nanoparticles and astrocyte/endothelial interactions
Source: F1000Res. 2016 Jan 21;4:1279. Originally published 2015 Nov 17. [Version 2] doi: 10.12688/f1000research.7142.2 (PMC4732555; doi:10.12688/f1000research.7142.2)
Supplement: Peer review history

# Peer review: Referee response for version 2

**DOI:** 10.5256/f1000research.8385.r12033

**Published:** 2016-01-22

**Authors:** Christopher C.W. Hughes

**Affiliations:** 1 Department of Molecular Biology and Biochemistry, University of California, Irvine, Irvine, CA, USA

While I still believe that the presence of pericytes would be useful and informative I do understand the authors' arguments about reducing complexity.

I have read this submission. I believe that I have an appropriate level of expertise to confirm that it is of an acceptable scientific standard.

# Peer review: Referee response for version 2

**DOI:** 10.5256/f1000research.8385.r12032

**Published:** 2016-01-22

**Authors:** Abraham J. Al Ahmad

**Affiliations:** 1 Department of Pharmaceutical Sciences, Texas Tech University Health Sciences Center, Amarillo, TX, USA

All the concerns and comments that were mentioned in the first version have been addressed.

I have read this submission. I believe that I have an appropriate level of expertise to confirm that it is of an acceptable scientific standard.

# Peer review: Referee response for version 1

**DOI:** 10.5256/f1000research.7694.r11245

**Published:** 2015-12-21

**Authors:** Christopher C.W. Hughes

**Affiliations:** 1 Department of Molecular Biology and Biochemistry, University of California, Irvine, Irvine, CA, USA

There is a tremendous demand for a reproducible and representative model of the human blood brain barrier and many versions have been tried. This paper represents an interesting variation, especially in the dispensing of transwells, which impose an unacceptable separation between the endothelial cells and the astrocytes.

There are a few issues I would like to see addressed, either by experimentation or additional discussion:

 

1. Pericytes are also an important component of the microvasculature and their addition here would likely strengthen the relevance of the model.
2. Given the importance of astrocytes in promoting development of the BBB it would be interesting to know whether they modulate additional markers of the BBB, such as ABC transporters, other than ZO-1 and Claudin 5 (both of which are only weakly induced).
3. The failure to induce pgp and the relatively high leakiness of the monolayer are  concerns as pgp expression and a tight barrier are both key features of the BBB *in vivo*.
4. Was a non-binding antibody used for the negative control in IF experiments? If so, this should be stated.
5. AQP4 is reduced in overall expression in 3D culture, but is it relocalized to the contact points between the astrocytes and the EC. This could be checked by confocal microscopy.
6. In Fig. 6, the arrows are missing.
7. In Fig. 7, “J” and “E” are missing from the figures.

I have read this submission. I believe that I have an appropriate level of expertise to confirm that it is of an acceptable scientific standard, however I have significant reservations, as outlined above.

# Response

**Published:** 2016-01-11

**Authors:** David Male

**Affiliations:** The Open University, UK

1. We agree with the reviewer that incorporating pericytes will make the model more relevant. Some previous studies ( Al Ahmad, 2010) have included the pericytes in 3D collagen gels. It would be closer to the in vivo conditions if we could grow a layer of pericytes between the endothelial monolayer and 3D cultured astrocytes. However, this will increase the complexity of the model, so to keep the model simple and suitable to study transcytosis, we decided to include only two cell types - the brain endothelium which must be crossed by nanoparticles and one example of a target cell, the astrocytes. 

2. ABC transporter B1 also known as P-gp-1 is expressed in hCMEC/D3 at similar levels to primary brain endothelium and was found not to be regulated by either 2D cultured or 3D cultures astrocytes. These results are presented in figure 4.

3.  Although it is highly desirable that *in vitro *modesl should simulate all aspects of the blood brain barrier *in vivo*, this has not been possible with any models produced to date. In this case we make it clear where the model is less useful (tight barrier properties and permeability for small molecules) but it is nevertheless useful for the functions indicated - nanoparticle transcytosis and targeting to astrocytes and leukocyte transendothelial migration.

4.  Immunofluorescence : For mouse monoclonal antibodies, the corresponding isotype immunoglobulin was used as control whereas for rabbit polyclonal antibodies only antibody diluent was used as control. This has been consistently followed in this study because we found that normal rabbit IgG produced a weak non-specific binding, increasing the image backgrounds.

5. We agree with the referee and as mentioned in the main text, the model allows analysis of local contacts between the astrocytes and endothelium by fluorescence or electron microscopy, which is an advantage over conventional co-cultures. However, since the astrocyte density was always <10% of the gel volume the contact area between astrocytes and endothelium was less than 10% of the endothelial monolayer, number of contacts were less. Within these limited observations, we did not see  relocalization of the AQP4 to the contacting end feet of astrocyte.

6/7 Additional annotations made to figures.

# Peer review: Referee response for version 1

**DOI:** 10.5256/f1000research.7694.r11397

**Published:** 2015-12-16

**Authors:** Abraham J. Al Ahmad

**Affiliations:** 1 Department of Pharmaceutical Sciences, Texas Tech University Health Sciences Center, Amarillo, TX, USA

The manuscript sounds technically good and the results were innovative. However, I have some recommendation or comments that would make bring this study to its highest potential.

1. Figure 2: A Claudin-5 immunostaining would be appreciated, especially to observe how the presence of astrocytes may improve claudin-5 immunoreactivity in hCMEC/D3 cells. What happens to adherent junctions such as VE-cadherin, beta-catenin or PECAM-1? Having such immunocytochemistry data in addition would greatly help.
  A GFAP staining would be appreciated.
2. Figure 4: I have some concerns about the absence of error bars in P-gp control group, although CLDN5 and ZO1 control groups display one. Please keep it consistent.
3. Figure 7: A true negative control such as TE7 fibroblasts is needed.

I have read this submission. I believe that I have an appropriate level of expertise to confirm that it is of an acceptable scientific standard, however I have significant reservations, as outlined above.

# Response

**Published:** 2016-01-11

**Authors:** David Male

**Affiliations:** The Open University, UK

1. We have previously shown that Claudin-5 immunostaining on hCMEC-D3 cells is more patchy than ZO-1 staining (Weksler et al, 2005). This may partly account for the relatively low trans-endothelial resistance of this cell line. Cnsequently claudin-5 staining was not done. Data in figure 4 suggests that claudin-5 is induced by astrocytic factors  although there was no significant difference between 2D and 3D  culture factors. PECAM was unchanged in solo-culture and co-culture (data not presented) and VE-cadherin was not investigated. A GFAP stain has been added to supplementary material.

2. In figure 4 Pgp was measured by flow cytometry with data adjusted to the untreated cells on each experiment (=100%). For this reason there are no error bars on the untreated cells. For complete understanding of how the data was normalized see dataset 4.

3. A negative control image has been added to the supplementary material.
